# Age-related alteration in characteristics, function, and transcription features of ADSCs

**DOI:** 10.1186/s13287-021-02509-0

**Published:** 2021-08-23

**Authors:** Keya Li, Guiying Shi, Xuepei Lei, Yiying Huang, Xinyue Li, Lin Bai, Chuan Qin

**Affiliations:** Key Laboratory of Human Disease Comparative Medicine, Chinese Ministry of Health, Beijing Key Laboratory for Animal Models of Emerging and Reemerging Infectious Diseases, Institute of Laboratory Animal Science, Chinese Academy of Medical Sciences and Comparative Medicine Center, Peking Union Medical College, No.5 Panjiayuan Nanli, Chaoyang District, Beijing, 100021 People’s Republic of China

**Keywords:** ADSCs, Autologous stem cell transplantation, Aging, Chemokine, Cell cycle, Cell culture

## Abstract

**Background and objectives:**

Adipose tissue-derived stem cells (ADSCs) autologous transplantation has been a promising strategy for aging-related disorders. However, the relationship between ADSCs senescence and organismal aging has not been clearly established. Therefore, we aimed at evaluating senescence properties of ADSCs from different age donors and to verify the influence of organismal aging on the proliferation and function of ADSCs in vitro, providing the theoretical basis for the clinical application of autologous ADSCs transplantation.

**Methods and results:**

The ADSCs were obtained from 1-month-old and 20-month-old mice. The cells characteristics, functions, gene expression levels, apoptosis proportion, cell cycle, SA-β-gal staining, and transcription features were evaluated. Compared to ADSCs from 1-month-old mice, ADSCs from 20-month-old mice exhibited some senescence-associated changes, including inhibited abilities to proliferate. Moreover, differentiation abilities, cell surface markers, and cytokines secreting differed between 1M and 20M ADSCs. SA-β-Gal staining did not reveal differences between the two donor groups, while cells exhibited more remarkable age-related changes through continuous passages. Based on transcriptome analysis and further detection, the CCL7-CCL2-CCR2 axis is the most probable mechanism for the differences.

**Conclusions:**

ADSCs from old donors have some age-related alterations. The CCL7-CCL2-CCR2 axis is a potential target for gene therapy to reduce the harmful effects of ADSCs from old donors. To improve on autologous transplantation, we would recommend that ADSCs should be cryopreserved in youth with a minimum number of passages or block CCL7-CCL2-CCR2 to abolish the effects of age-related alterations in ADSCs through the Chemokine signaling pathway.

**Supplementary Information:**

The online version contains supplementary material available at 10.1186/s13287-021-02509-0.

## Background

Aging is a multifactorial phenomenon and an extremely complex process that affects the biological functions of an organism, generally culminating in disease and death due to the accumulated actions of different types of stresses [1, 2]. Age-associated pathologies, including neurodegenerative diseases, cardiovascular disorders, and certain metabolic diseases, are common diseases associated with the aging process that can severely affect patients’ quality of life and represent a major economic challenge for families and the society in general [3]. With the growing aging population, disorders associated with cognitive and behavioral dysfunctions are becoming a public health concern [4].

Autologous stem cell transplantation is an effective therapeutic option that has been used as a standard first-line therapy for plasma cell dyscrasia, Parkinson’s disease, and amyotrophic lateral sclerosis (ALS) patients [5–8], which could eliminate the post-engrafting immunological rejection. With less ethical concerns when used, adult stem cells are the main source of autologous transplantation which maintains tissue homeostasis [9]. Zuk first isolated and identified adipose tissue-derived stem cells (ADSCs) from adipose tissue in 2002 [10, 11], and since then, studies on ADSCs have rapidly increased. Due to their plasticity and to the fact that they can be easily collected by minimally invasive surgical techniques, ADSCs are advantageous for therapeutic applications [12, 13]. ADSCs have an enormous potential in vitro amplification and differentiative capacity into several specific cells like neuronal cells, muscle epithelial cells, cardiac myocytes, osteoblasts, and chondrocytes [14–20]. Cells express mesenchymal cell lineage markers, including CD90, CD29, CD73, CD105, and CD44, and do not express hematopoietic lineage markers, such as CD45, CD34, and CD11b, or the endothelial cell marker CD31 [21–24]. Due to the absence of human leukocyte antigen (HLA)-DR expression, ADSCs are relatively immune-privileged mesenchymal stem cells (MSCs) that can be used as effective therapies for providing graft versus host disease (GvHD) patients [25]. Moreover, ADSCs exhibit immunosuppressive, anti-apoptosis, anti-oxidation, as well as anti-inflammatory properties and secrete various cytokines [26, 27]. A dominant cytokine secreted by ADSCs is interleukin 6 (IL-6), which is a pleiotropic cytokine. This cytokine is involved in the immune responses and it influences the formation of new blood vessels by regulating the expression of vascular endothelial growth factor (VEGF) [28, 29].

As a promising source of autologous cells with lower immunogenicity for the therapy of neurodegenerative diseases, treatment outcomes associated with using young and aged ADSCs have not been established in concrete aspects. Moreover, the relationships between aged ADSCs and senescence have not been conclusively determined. It has been reported that aged ADSCs have a significantly compromised in their ability to support vascular network formation [30]. Schultz argued that with the onset of senescence, the number of cells and their abilities decrease over, while stem cell pools maintain good characteristics even as several stem cells are gradually depleted [31]. Schipper reported that the production, proliferative rate, and pluripotency of adipose-derived stem cells among young individuals are superior to those of the elderly [32]. Alt confirmed that the expression of senescence genes of adipose-derived stem cells increase with age, while the miRNA, which is associated with cell cycle regulation, apoptosis, and the ability to maintain homeostasis decreased [33]. Shi [34] and Zhu [35] documented that age does not affect the ability of ADSCs to proliferate and differentiate. Aust [36] confirmed that the senescence of ADSCs is not correlated with age while Mojallal [37] proved that the yield and proliferative capacities of ADSCs are not correlated with age.

To establish the correlation between aging and cell senescence, we isolated ADSCs from 1-month-old mice and 20-month-old mice and analyzed their characteristics of ADSCs. The morphologies, ultrastructures, proliferation, differentiation, functions of secreting cytokines, surface markers, apoptosis, cell cycle, senescence-associated β-galactosidase (SA-β-gal) staining, and gene expression levels of ADSCs from the two age groups were determined and compared. To investigate the underlying mechanism of these differences, we evaluated the two groups of mice ADSCs (mADSCs) through transcriptome sequencing (RNA-seq).

## Methods

### Study animals

We purchased 1-month-old SPF-grade C57BL/6 mice from Beijing HFK Bio-Technology Co., Ltd., while the 20-month-old SPF-grade C57BL/6 mice were obtained from the experimental animal workshop. All animal experiments were performed according to the accepted standards of animal care. Ethical approval for the use of animals in this study was obtained from the Animal Care and Use Committee of the Institute of Laboratory Animal Science of Peking Union Medical College (No.BL17001).

### Isolation of ADSCs

To explore the effects of donor age on ADSCs, we randomly isolated ADSCs from a stromal-vascular cell fraction (SVF) derived from the abdominal subcutaneous adipose tissue of C57 mice. Adipose tissue was washed twice in phosphate buffer saline (PBS, Gibco, USA) and digested with 0.075% collagenase in PBS at a volume ratio of 1:2 in a 37 °C water bath for 1 h. We added fetal bovine serum (FBS, Gibco, USA) to neutralize them and centrifuged at 400×*g*/min for 11 min, and then discarded the supernatant. Red blood cells were lysed in Tris-NH4Cl (Solarbio, China) at room temperature for 5 min and centrifuged at 400×*g*/min for 5 min after the suspension through 70 μm filter, and then the supernatant was discarded. Lastly, cells seeded on 25 cm^2^ tissue culture flasks in Dulbecco’s modified Eagle’s medium (DMEM, Gibco, USA) containing 10% FBS and 1% penicillin-streptomycin (Gibco, USA) and cultured in a humidified atmosphere at 37 °C, 5% CO_2_. The medium was replaced every third day. When ~ 80% confluency was reached, cells were detached using 0.25% Trypsin-EDTA (Gibco, USA).

### Morphology of ADSCs

ADSCs were observed by a phase-contrast microscope (Leica, German). And the ultrastructure was observed by transmission electron microscope (JEM-1400, Japan). Cell surface area was calculated with ImageJ (version 2.0.0, MD, USA). Cells were thresholded to remove the background to identify the whole-cell region of interest, and the area of each cell was measured and plotted.

### CCK-8 assay for cell proliferation

Cell proliferation was evaluated using the CCK-8 kit (Beyotime, China). Briefly, 100 μL of 1,000 cells were added to each well of the 96-well plate. After the cells adherence, 10 μL of the CCK-8 solution was directly added to each well and incubated at 37 °C in a 5% CO_2_ atmosphere for 1 h. On day 0, absorbance was measured at 450 nm. Then, after 24 h of incubation (day 1), absorbance was measured again, as was done on days 2, 3, 4, 5, and 6. Finally, based on OD values, a growth curve was drawn.

### Flow cytometry

ADSCs were harvested in the third passage (P3), washed in cold PBS, incubated with APC-conjugated rat anti-mouse CD105 (clone MJ7/18, eBioscience), PE-conjugated hamster anti-mouse CD29 (HMb1-1, eBioscience), FITC-conjugated rat anti-mouse CD34 (RAM34, eBioscience), PE-eFluor780-conjugated rat anti-mouse CD11b (M1/70, eBioscience), and PE-conjugated rat anti-mouse CD45 (clone 30-F11, BD) for 30 min from no light at 4 °C. Flow cytometry data were collected with the acquisition of 10,000 events per sample using the BD FACSAriaII machine (BD Biosciences, USA). Gate P2 was set according to the staining intensity of appropriate isotype-matched control antibodies. FASCDiva software (BD Biosciences, USA) and FlowJo software (FlowJo LLC, Ashland, Oregon) were used for data analysis.

### Adipogenic and osteogenic differentiation

For adipogenesis, ADSCs were cultivated in 24-well culture dishes until 100% confluent. Regular culture medium was replaced with adipogenesis induction medium (Cyagen Biosciences, USA). Cells in control wells were cultivated in basic mediums. Lipid droplets were detected after 14 days by Oil Red O staining. Oil Red O was dissolved in 200 μL 100% isopropanol (Beijing Chemical Works, China) and incubated at room temperature for 5 min, and the content of dissolution was measured by spectrophotometer at OD 495 nm. For osteogenesis, ADSCs seeded on 24-well plates were allowed to reach 70% confluency. Osteogenic differentiation was induced by adding osteogenic induction mediums (Cyagen Biosciences, USA). Control cells were cultivated in basic mediums. After 21 days of cultivation, extracellular calcium accumulation was detected by Alizarin Red S. To quantify the calcium deposits, three random fields with × 100 magnification were selected. The relative area of Alizarin Red S stain positivity within each group was calculated and quantified by Image J software.

### Senescence-associated β-galactosidase (SA-β-gal) staining

To evaluate cellular senescence, ADSCs were fixed and stained with senescence-associated β-galactosidase (SA-β-Gal) staining kit (Beyotime, China) at 37 °C for 16 h. To quantify the senile cells, three random fields with × 200 magnification were selected. The cells positively stained with β-Gal were quantified by Image J software.

### Cytokine array

A mouse cytokine array kit was used for simultaneous detection of 62 cytokines according to the manufacturer’s protocol (Abcam, USA). Briefly, the P3 cell culture, which had been incubated for 24 h, was added to the membrane of a mouse cytokine array. After washing the membrane, a detection antibody was added and immunoblot images were captured using the ChemiDoc™ XRS+ System (Bio-Rad, USA). The intensity of each spot was measured using the Image J software (version 2.0, Maryland, USA).

### Next-generation RNA sequencing (RNAseq)

#### Sample collection and preparation

##### RNA quantification and qualification

RNA degradation and contamination were monitored on 1% agarose gels. RNA purity was checked using the NanoPhotometer® spectrophotometer (IMPLEN, CA, USA). RNA concentration was measured using Qubit® RNA Assay Kit in Qubit® 2.0 Fluorometer (Life Technologies, CA, USA). RNA integrity was assessed using the RNA Nano 6000 Assay Kit of the Bioanalyzer 2100 system (Agilent Technologies, CA, USA).

##### Library preparation for transcriptome sequencing

A total amount of 3 μg RNA per sample was used as input material for the RNA sample preparations. Sequencing libraries were generated using NEBNext® UltraTM RNA Library Prep Kit for Illumina® (NEB, USA) following the manufacturer’s recommendations, and index codes were added to attribute sequences to each sample. At last, PCR products were purified (AMPure XP system), and library quality was assessed on the Agilent Bioanalyzer 2100 system.

##### Clustering and sequencing

The clustering of the index-coded samples was performed on a cBot Cluster Generation System using TruSeq PE Cluster Kit v3-cBot-HS (Illumina) according to the manufacturer’s instructions. After cluster generation, the library preparations were sequenced on an Illumina Hiseq platform and 125 bp/150 bp paired-end reads were generated.

#### Data analysis

##### Quality control

Raw data (raw reads) of fastq format were firstly processed through in-house perl scripts. In this step, clean data (clean reads) were obtained by removing reads containing adapter, reads containing ploy-N, and low-quality reads from raw data. At the same time, Q20, Q30, and GC content of the clean data were calculated. All the downstream analyses were based on clean data with high quality.

##### Quantification of gene expression level

HTSeq v0.6.0 was used to count the reads numbers mapped to each gene. And then FPKM of each gene was calculated based on the length of the gene and the reads count mapped to this gene. FPKM, the expected number of fragments per kilobase of transcript sequence per million base pairs sequenced, considers the effect of sequencing depth and gene length for the reads count at the same time and is currently the most commonly used method for estimating gene expression levels.

##### Differential expression analysis

Before differential gene expression analysis, for each sequenced library, the read counts were adjusted by the edgeR program package through one scaling normalized factor. Differential expression analysis of two conditions was performed using the edgeR R package (3.12.1). The P values were adjusted using the Benjamini and Hochberg method. Corrected *P* value of 0.05 and absolute foldchange of 2 were set as the threshold for significantly differential expression.

##### GO and KEGG enrichment analysis of differentially expressed genes

Gene Ontology (GO) enrichment analysis of differentially expressed genes was implemented by the clusterProfiler R package, in which gene length bias was corrected. GO terms with corrected *P* value less than 0.05 were considered significantly enriched by differential expressed genes.

KEGG is a database resource for understanding high-level functions and utilities of the biological system, such as the cell, the organism, and the ecosystem, from molecular-level information, especially large-scale molecular datasets generated by genome sequencing and other high-throughput experimental technologies (http://www.genome.jp/kegg/). We used clusterProfiler R package to test the statistical enrichment of differential expression genes in KEGG pathways.

##### GSEA and NTA of differentially expressed genes

Gene set enrichment analysis (GSEA) and network topology-based analysis (NTA) of differentially expressed genes was performed using the WEB-based Gene SeT AnaLysis Toolkit (http://www.broad.mit.edu/GSEA/) [38]. Parameters set as significance level at FDR< 0.05.

### Quantitative RT-PCR

Gene expression levels in ADSCs obtained from young and aged mice were evaluated by quantitative real-time RT-PCR using SYBR Green Premix Ex Taq II (Tli RNaseH Plus). Briefly, total RNA was extracted using the Qiagen RNeasy Mini kit (Qiagen, Valencia, CA), according to the manufacturer’s instructions. cDNA was synthesized using the cDNA archive reverse transcription kit (Life Technologies), according to the manufacturer’s instructions. Primers for *p16*, *p19*, *p21*, *Ddx3y*, *S100a9*, *S100a8*, *Ngp*, *Chil3*, *Lef1*, *Mcpt1*, *Dthd1*, *Ajap1*, *Kcnmb4*, *Myc*, *Axin2*, *Taz*, *Yap1*, *Ccl7*, *Ccl2*, *Ccr2*, and *β-actin* were purchased from ThermoFisher (Table S1). Beta actin was used as the reference gene for normalization. Gene expression levels were quantified by the cycle threshold (Ct) method (ΔΔCt).

### Statistical analysis

Mean and SE was calculated by averaging the results of three to six independent experiments performed with independent adipose cultures obtained from the individual mouse for each experiment. The SPSS software (version 25, IL, USA) was used to determine significant differences between the means. *p* < 0.05 was considered significant. ∗*p* < 0.05.∗∗*p* < 0.01. ∗∗∗*p* < 0.001. ∗∗∗∗*p* < 0.0001.

## Results

### Aged ADSCs have the ultrastructure, cell proliferation, and cytokines secreting alteration

**Fig. 1:**
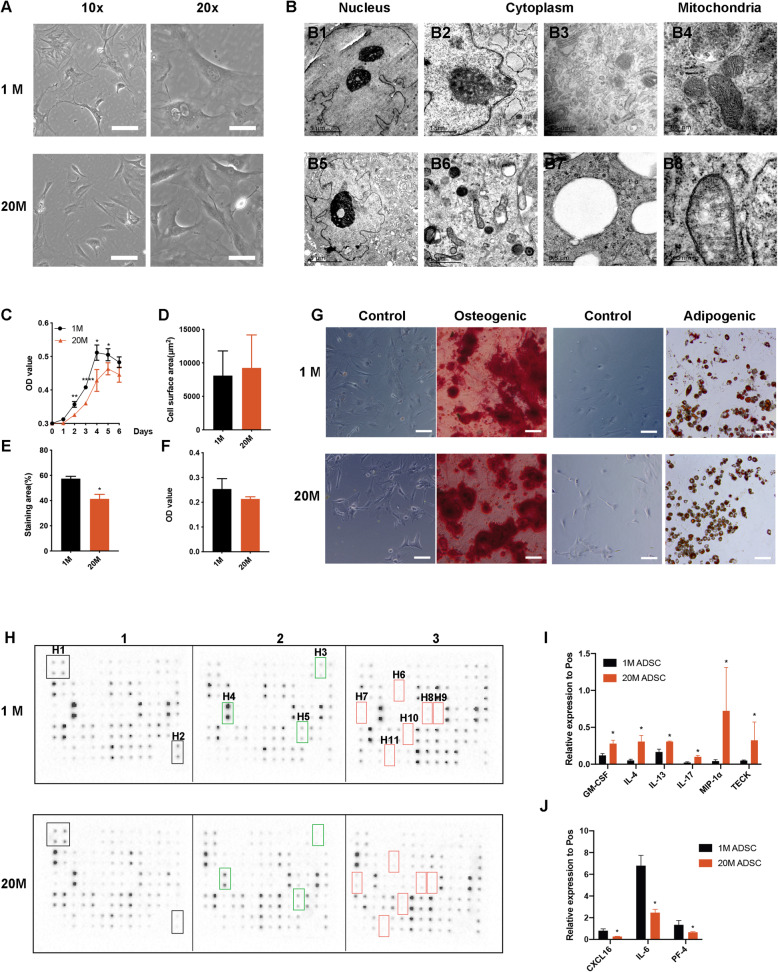
Morphologies and functions of ADSCs from 1M and 20M mice. **A** ADSCs morphologies under an optical microscope. Scale bars of 10× groups, 100 μm. Scale bars for 20× groups, 75 μm. **B** Ultrastructures of ADSCs under a transmission electron microscope (TEM). B1-B4 represent ADSCs from 1-month-old (1M) mice while B5–B8 represent ADSCs from 20-month-old (20M) mice. **C** Growth curves of ADSCs. ∗*p* < 0.05. ∗∗*p* < 0.01. ∗∗∗∗*p* < 0.0001. **D** Cell surface areas of ADSCs. **E** Osteogenic differentiation levels of ADSCs from 1M and 20M mice. Scale bars, 100 μm. **F** OD values of Oil Red O stainings of ADSCs from 1M and 20M mice. **G** Osteogenic and adipogenic differentiation capacities of 1M and 20M ADSCs. Scale bars, 100 μm. **H** Cytokine assay of ADSCs from 1M and 20M ADSCs. H1 and H2, positive controls. H3-H5, CXCL-16, IL-6, and PF-4, respectively. H6-H11, GM-CSF, IL-4, IL-13, IL-17, CCL3, and CCL25, respectively. ∗*p* < 0.05. **I** Relative expression of GM-CSF, IL-4, IL-13, IL-17, MIP-1α, and TECK as 1M ADSCs was control. ∗*p* < 0.05. **J** Relative expression of CXCL16, IL-6, and PF-4 as 1M ADSCs was control. ∗*p* < 0.05

To assess cellular senescence in ADSCs, cell cultures from five 1-month-old mice and five 20-month-old mice were in vitro cultivated. After 7 days, most cells had adhered to the tissue culture plate, assuming a fibroblast-like phenotype as flat and spindle-shaped cells. There were no morphological differences in ADSCs obtained from the aged and young mice, as determined by phase contrast microscopy (Fig. 1 A and D).

Then, ADSCs were fixed to evaluate their inner structures and cell composition. It was found that they exhibited large and round cell bodies with pseudopodia. Homogeneous round lipid droplets and a lot of ribosomes were observed in the cytoplasm. The ADSCs were shown to have a lobular or polygonal large nucleus and a high karyoplasmic ratio. In addition, there were abundant projections and depressions on the nuclear membrane, as well as 1-3 nucleoli in the nucleus. Upon further magnification view, the mitochondria in the cytoplasm exhibited a bilayer membrane structure, with the inner membrane folded into cristae. Compared to ADSCs from 1-month-old mice (1M ADSCs), ADSCs from 20-month-old mice (20M ADSCs) showed swollen rough endoplasmic reticulums, a large number of medullary corpuscles in their cytoplasms (Fig.1B, B6), and an increased abundance of lipid droplets (Fig.1B, B7). Moreover, the mitochondria were swollen with altered crest structure in old group (Fig. 1B, B8).

When cultured to P3, cells were seeded in a 24-well plate at a density of 1×10^3^ per well and measured the OD value every day. Then, growth curve of ADSCs from 1M and 20M mice were drawn. Figure 1C shows that, compared to the 20M ADSCs, the 1M ADSCs entered the exponential growth phase within a shorter period of time, implying a fast growth rate.

To determine functional differences, differentiation capacities of 1M and 20M ADSCs into the adipogenic and osteogenic lineages were evaluated. It was found that 1M and 20M ADSCs were capable of osteogenic and adipogenic differentiation, with 1M ADSCs exhibiting stronger osteogenesis (Fig. 1E–G). In addition, cytokine secretory functions of the cells were determined by a cytokine array. All cytokine targets were shown listed in Table S2. Remarkably, there were greater fold changes for downregulated cytokines (Fig.1H). Moreover, 29 of the 62 cytokines were upregulated, with granulocyte-macrophage colony-stimulating factor (GM-CSF), interleukin-4 (IL-4), IL-13, IL-17, macrophage inflammatory protein-1α (MIP-1α) or C-C motif chemokine ligand 3 (CCL3), and thymus-expressed chemokine (TECK) or CCL25 being significantly expressed (Fig.1I and Fig. S1B). Three out of the residual 33 cytokines, including C-X-C Motif Chemokine Ligand 16 (CXCL-16), IL-6, and Platelet Factor 4 (PF-4), were found to be significantly downregulated (Fig.1J and Fig. S1C).

### Apoptotic rates were increased in 20M ADSCs accompanied by increased number of cells in the quiescent G0 phase

**Fig. 2:**
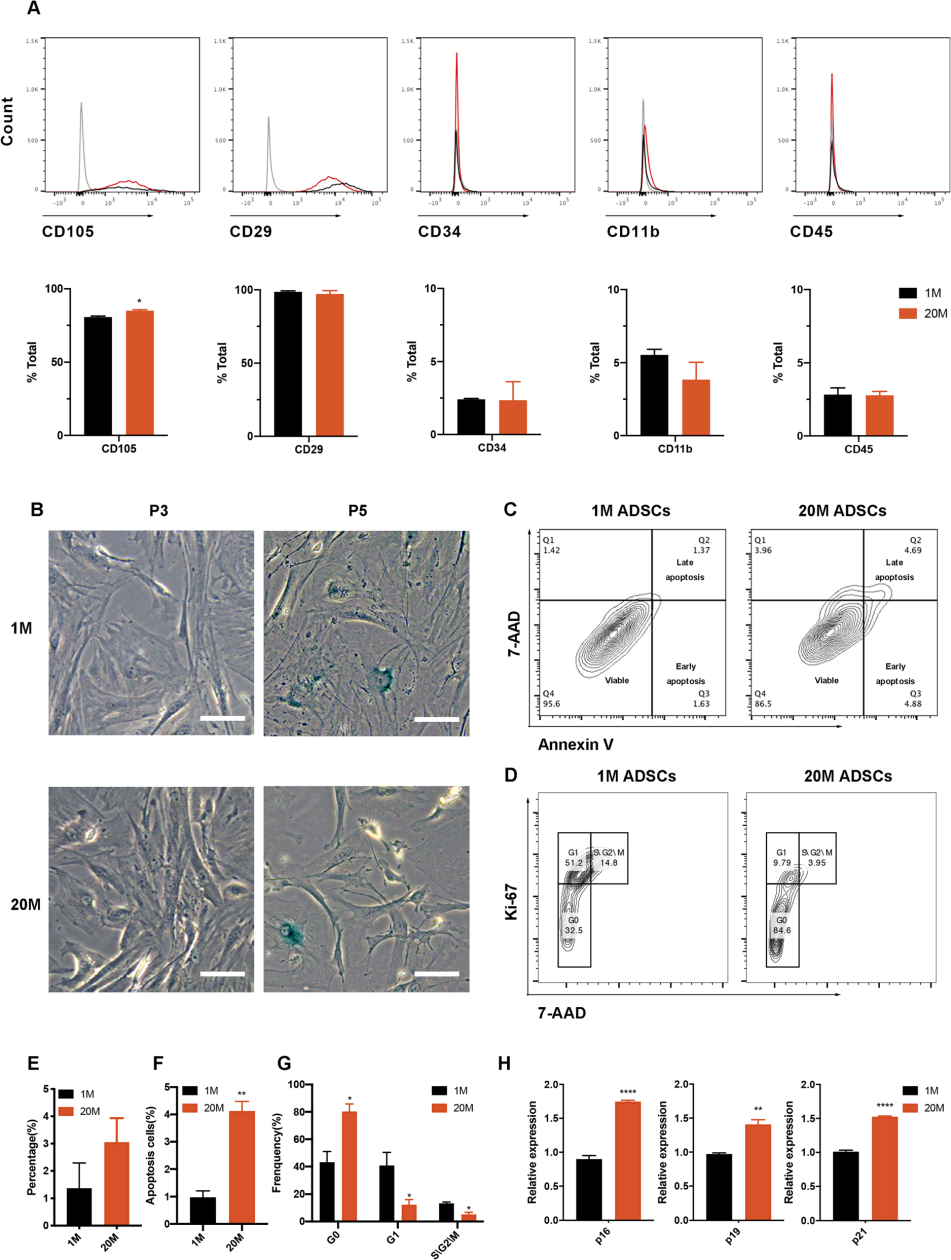
Surface markers and age-related phenotypes of ADSCs. **A** Proportions of ADSCs expressing positive and negative surface markers. **B**, **E** SA-β-gal staining of 1M and 20M ADSCs. Scale bars, 100 μm. **C**, **F** Annexin V and 7-AAD between 1M and 20M ADSCs were analyzed by flow cytometry. ∗∗*p* < 0.01. **D**, **G** Flow cytometry analysis of 7-AAD and Ki-67 between 1M and 20M ADSCs. ∗*p* < 0.05. **H** Age-related gene expression levels in 1M and 20M ADSCs. ∗∗*p* < 0.01. ∗∗∗∗*p* < 0.0001

All the cells exhibited the ADSCs surface markers—positive in CD105 and CD29 and negative in CD34, CD11b, and CD45. Figure 2A shows that the average percentages of CD105, CD29, CD34, CD11b, and CD45 in 1M ADSCs were 80.7, 98.6, 2.41, 5.53, and 2.83, while the average percentage in 20M ADSCs were 85.0, 97.2, 2.36, 3.84, and 2.78, respectively. There were no significant differences in most of the surface markers between 1M and 20M ADSCs, implying that the adipose tissue obtained from 1-month-old mice had a comparative yield of ADSCs to that of 20M mice under the same conditions.

SA–β-Gal staining revealed a few senescence cells during early passages between the two types of ADSCs (Fig. 2B). After two serial cell passages every 3–4 days, the number of SA–β-Gal-positive cells were determined. There were no significant differences in the abundance of SA–β-Gal-positive cells between the two groups (Fig. 2B and E). The abundance of senescent cells were increased as the number of passages increased, indicating that passage time has a greater effect on senescence than donor age.

The influence of donor age on the cell cycle and apoptosis was measured by flow cytometry (Fig. 2C, D, F, and G). Compared to 1M ADSCs, there was an increase in the number of apoptotic cells in 20M ADSCs (Fig. 2C and F). Moreover, there was an increase in the number of 20M ADSCs in the G0 phase increase, while cells in G1 and S/G2/M phases were decreased, when compared to 1M ADSCs (Fig. 2D and G).

Finally, analysis of aging-related gene expression levels by qRT-PCR revealed elevated expression levels of p19 in 20M ADSCs, compared to 1M ADSCs. p16 and p21 are two markers that are frequently associated with cellular senescence [39], and their expression levels in 20M ADSCs were found to be elevated (Fig. 2H).

### Lef1 and Ddx3y respectively downregulated and upregulated in 20M ADSCs

To investigate the underlying mechanisms of these differences, we performed RNA-seq on ADSCs from 1M and 20M mice. Fig. S3 shows images of agarose gels on which the RNA degradation was monitored. RIN values of each sample are shown in Table S3. Sequencing yielded on average 39.2 million raw reads per 1M ADSCs and 33.5 million raw reads per 20M ADSCs. Supporting Information Table S4 shows that Q20 of 1M ADSCs and 20M ADSCs were more than 95%, while the Q30 was more than 90%. Alignment analysis revealed an average total read mapping rate of 95.51% and a proper pair alignment rate of 88.35% in 1M ADSCs, while 20M ADSCs had an average total read mapping rate of 95.39% and proper pair alignment rate of 88.26% (Table S5). RPKM values of the two groups are shown in Table S6. RPKM is a marker of significant differences in gene expressions when it is more than 1.

**Fig. 3:**
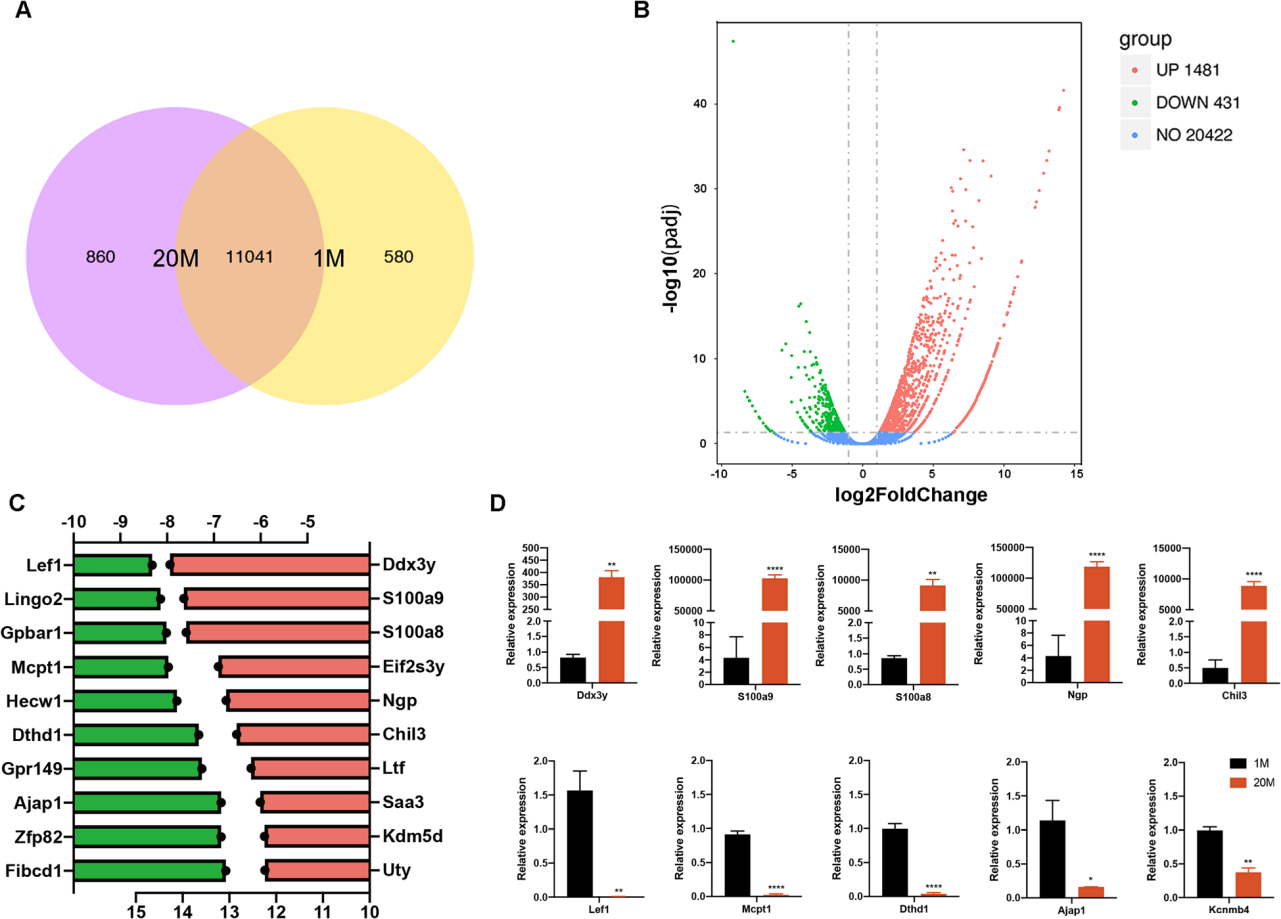
Differential gene expression analysis. **A** Venn chart showing 11041 differentially expressed genes (DEGs) that were common between 1M and 20M ADSCs. Corrected *P* value of 0.05 and absolute fold change of 2 was set as the threshold for significantly differential expression. **B** Volcano plot of DEGs that were significantly downregulated (green) and upregulated (red) compared to 1M ADSCs. **C** Profiles of the top 10 DEGs. Green bar indicates significantly downregulated DEGs, while red indicates significantly upregulated DEGs. **D** Verification of DEGs through qRT-PCR. ∗*p* < 0.05. ∗∗*p* < 0.01.∗∗∗∗*p* < 0.0001

1M and 20M ADSCs have 11,041 genes in common (Fig. 3A). Overall distributions of differentially expressed genes (DEGs) between the two groups were based on the values of fold changes and the significance levels, which could be inferred from the volcano plot. Compared to 1M ADSCs, DEGs from 20M ADSCs were colored in green and red, representing 431 significantly downregulated and 1481 significantly upregulated genes, respectively (Fig. 3B). The top 10 most downregulated genes were *Lef1*, *Lingo2*, *Gpbar1*, *Mcpt1*, *Hecw1*, *Dthd1*, *Gpr149*, *Ajap1*, *Zfp82*, and *Fibcd1* while the top 10 most upregulated genes were *Ddx3y*, *S100a9*, *S100a8*, *Eif2s3y*, *Ngp*, *Chil3*, *Ltf*, *Saa3*, *Kdm5d*, and *Uty*, as shown by the absolute value of log_2_Foldchange with age (Fig. 3C). The DEGs were verified by qRT-PCR, and the gene expression patterns were found to be consistent with transcriptome results (Fig. 3D).

### Chemokine signaling pathway and Hippo signaling pathways were the top potential signaling pathways, indicating that these two pathways are correlated with age-related alterations in mADSCs

**Fig. 4:**
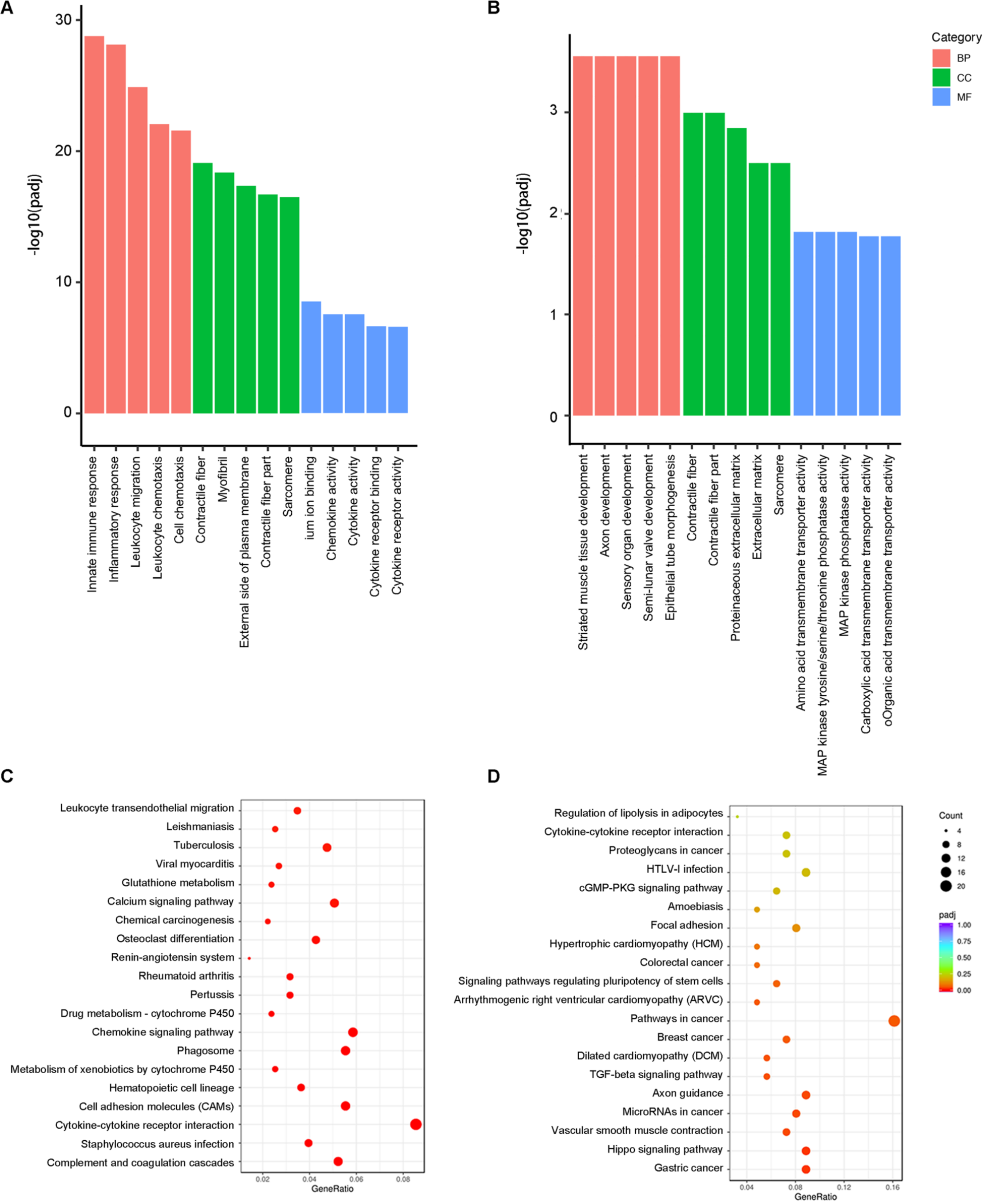
Enrichment results of upregulated and downregulated DEGs. **A**, **B** GO enrichment of age-related DEGs. The top five terms enriched for each category are shown. **A** Enrichment of upregulated genes. **B** Enrichment of downregulated genes. **C**, **D** KEGG pathway enrichment of age-related DEGs. The top twenty enriched pathways are shown. Count, differential gene count in the indicated pathway. padj, adjusted *p* value of the indicated pathway. GeneRatio, represented by the color scale, is the ratio of the number of genes in the dataset to the total number of genes in a pathway. **C** Upregulated genes enrichment; **D** Downregulated genes enrichment

To explore the changing profile of gene expression levels, upregulated and downregulated genes were analyzed separately. The DEGs were grouped into three GO categories: biological process (BP), cellular component (CC), and molecular function (MF). Upregulated DEGs were mostly associated with innate immune response, inflammatory response, leukocyte migration, leukocyte chemotaxis, and cell chemotaxis in BP. As for CC, the genes are mostly expressed differentially in contractile fiber and myofibril. Cytokine activity and chemokine activity are the most related terms of differentially expressed genes in MF (Fig. 4A). Figure 4C shows that KEGG analysis further identified cytokine-cytokine receptor interaction and chemokine signaling pathway as top-scoring pathways among the age-related upregulated genes (Fig.4C).

GO and KEGG pathway enrichment of age-related downregulated DEGs are shown in Figure 4B and D. GO analysis revealed that the DEGs were mainly enriched in striated muscle tissue development and axon development in BP, contractile fiber in CC, and amino acid transmembrane transporter activity in MF (Fig.4B). Seven KEGG pathways were significantly enriched, they were gastric cancer, Hippo signaling pathway, vascular smooth muscle contraction, microRNAs in cancer, axon guidance, TGF-beta signaling pathway, and dilated cardiomyopathy (DCM). KEGG enrichment levels were also measured using GeneRatio [40]. A higher GeneRatio indicates more gene targets belonging to a specific pathway [41]. Gastric cancer, Hippo signaling pathway, and axon guidance were the three pathways that were found to have the highest GeneRatio, with 11 out of 124 of the genes associated with aging (Fig. 4D).

### The chemokine signaling pathway was the most enriched in mADSCs with age

**Fig. 5:**
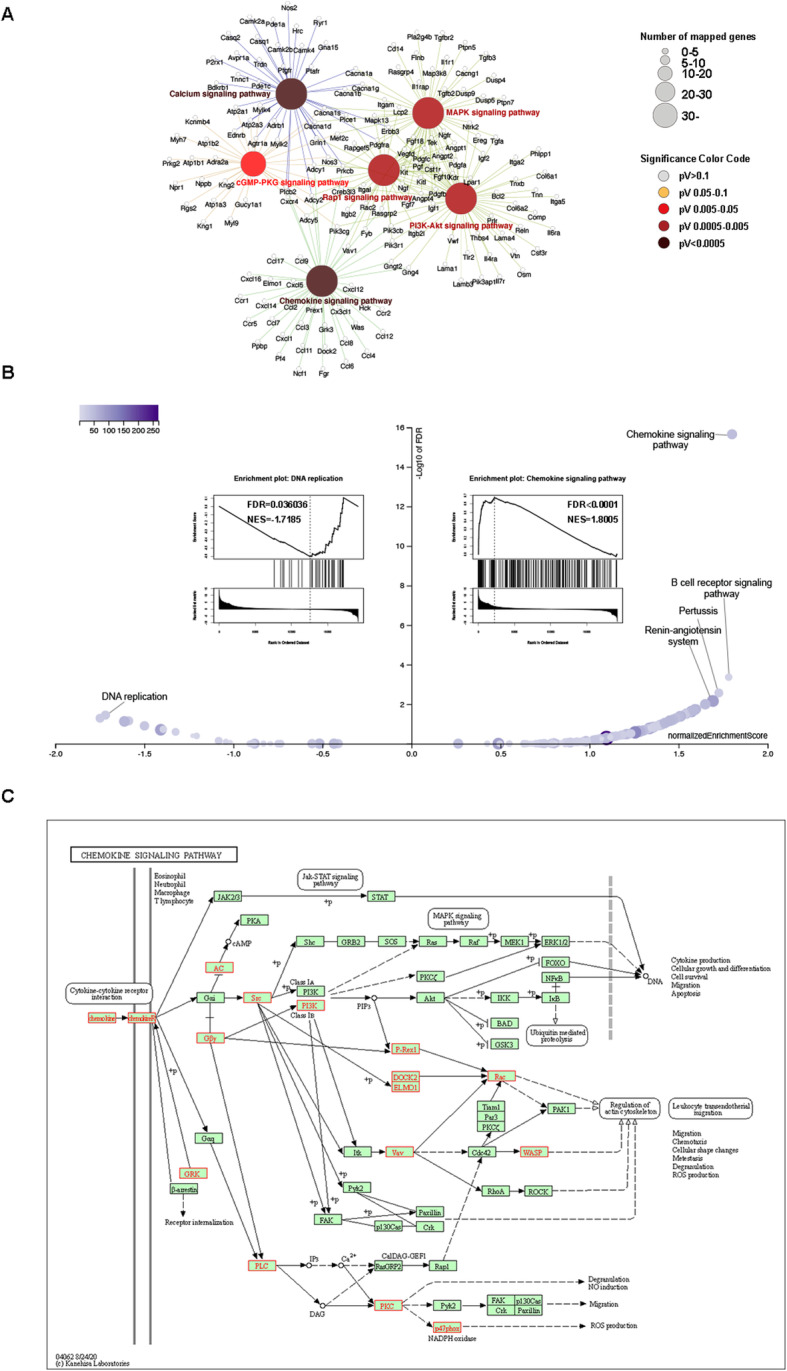
Enrichment of overall DEGs. **A** KEGG pathway enrichment of age-related DEGs. **B** Volcano and enrichment plot of GSEA in significance level at FDR < 0.05. NES, normalized enrichment score. FDR, false discovery rate. **C** Map of chemokine signaling pathway in the KEGG database. Mapped DEGs are shown in red

Then, GO and KEGG pathway enrichment analyses were performed overall on the enriched genes. Functional categorization revealed that most of the DEGs with age were associated with inflammatory response, contractile fiber, and calcium ion binding (Table 1). Overall, the DEGs were found to be associated with the following signaling pathways: Chemokine, Calcium, Rap1, PI3K-Akt, MAPK, and cGMP-PKG (Fig. 5A).

**Table 1:** GO functional annotation of overall DEGs

Category	GO ID	Description	GeneRatio	Padj	Up	Down
BP	GO:0006954	Inflammatory response	144/1636	1.08E-26	130	14
	GO:0045087	Innate immune response	135/1636	1.62E-25	126	9
	GO:0003012	Muscle system process	103/1636	2.47E-23	86	17
	GO:0006935	Chemotaxis	123/1636	2.47E-23	100	23
	GO:0050900	Leukocyte migration	89/1636	2.47E-23	83	6
CC	GO:0043292	Contractile fiber	82/1676	5.36E-26	66	16
	GO:0030016	Myofibril	75/1676	2.13E-23	62	13
	GO:0044449	Contractile fiber part	73/1676	2.78E-23	58	15
	GO:0030017	Sarcomere	68/1676	4.63E-22	55	13
	GO:0009897	External side of plasma membrane	77/1676	1.09E-18	68	9
MF	GO:0005509	Calcium ion binding	112/1622	1.43E-12	89	23
	GO:0008009	Chemokine activity	18/1622	5.38E-09	16	2
	GO:0005125	Cytokine activity	41/1622	1.92E-08	36	5
	GO:0030414	Peptidase inhibitor activity	39/1622	4.59E-08	33	6
	GO:0005126	Cytokine receptor binding	54/1622	5.04E-08	46	8

*Abbreviations*: GeneRatio, differential gene count in the indicated pathway versus total differential gene count; Padj, adjusted *p* value of the indicated pathway; Up, upregulated differential gene count in the indicated pathway; Down, downregulated differential gene count in the indicated pathway

To identify the significantly enriched signaling pathway from an overall perspective, a gene set analysis was performed at significance level at FDR < 0.05. A total of 49 positively related categories and 1 negative related category were identified as enriched categories. The 5 most significant categories and representatives in the reduced sets are shown in Fig. 5B. Clustering analysis revealed the that chemokine signaling pathway was clustered with the highest normalized enrichment score (NES, 1.8068) while the NES of the negatively related category (DNA replication) was − 1.7139. In the map of chemokine signaling pathway, enriched genes are shown in red (Fig. 5C, Table S6). Screened differentially expressed genes were imported into the PANTHER tool for pathway enrichment analysis. Inflammation mediated by chemokine and cytokine signaling pathway was the most significant positive category (Fig. S4).

### The CCL7-CCL2-CCR2 axis regulates age-related changes in mADSCs

**Fig. 6:**
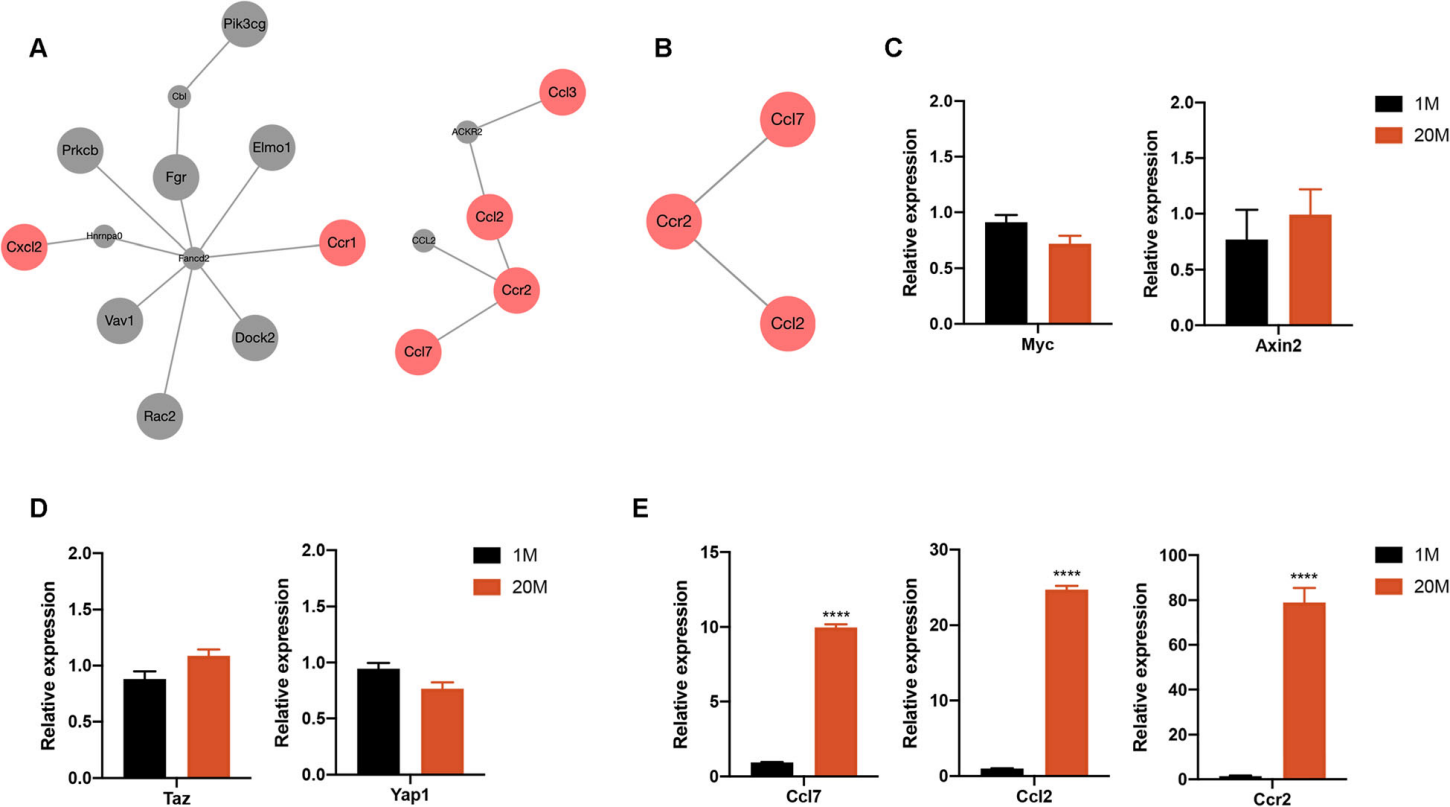
PPI network of DEGs mapped chemokine signaling pathway. **A** Expanded PPI network of DEGs mapped chemokine signaling pathway. **B** Retrieved PPI network of DEGs mapped chemokine signaling pathway. **C** mRNA expression levels of key genes involved in the Wnt signaling pathway in 1M and 20M ADSCs. **D** The mRNA expression levels of key genes involved in the Hippo signaling pathway from 1M and 20M ADSCs. **E** The mRNA expression levels of *Ccl7*, *Ccl2*, and *Ccr2* in 1M and 20M ADSCs. ∗∗∗∗*p* < 0.0001

During the evaluation of the clusters enriched in chemokine signaling pathway for Biogrid data, it was found that the Biogrid PPI network had one node *Ccl2* of a very high degree corresponding to the chemokine. Enrichment analysis revealed a total of 18 genes in the expanded sub-network. Based on the probability of random walk method, the top 5 neighbors are shown in Table S7. All seeds and top-ranking neighbors in the expanded sub-network were enriched in the 5 GO BP categories (Fig. 6A; Fig. S5A). In the retrieved sub-network, 3 genes (*Ccl7*, *Ccl2*, *Ccr2*) were found to be enriched in the 5 GO BP categories (Fig. 6B; Fig. S5B).

Lef1 was found to be the most downregulated gene, as described previously, implying that the Wnt signaling pathway may be involved. Moreover, the Hippo signaling pathway was found to be the most significant signaling pathway in RNA-seq. To further determine the potential mechanism of the age-related changes in ADSCs, expression levels of key genes in the Wnt signaling and Hippo signaling pathways, along with *Ccl7*, *Ccl2*, and *Ccr2* were determined. A quantitative real-time PCR array revealed that expression levels of *Ccl7*, *Ccl2*, and *Ccr2* in 20M ADSCs were elevated from aged donors, while expression levels of the other key genes in the two pathways were not significantly different (Fig. 6C-E).

## Discussion

We investigated age-related differences in ADSCs from 1-month-old and 20-month-old mice to elucidate on ADSCs senescence and organismal aging as well as evaluate the possible involved mechanisms.

In this study, the abdominal subcutaneous adipose tissue was selected because it is the most commonly used source of ADSCs. Moreover, the adipose tissue is relatively easy to acquire, and large quantities of adipose cells are obtained through liposuction [42, 43]. Then, we evaluated the morphologies, ultrastructures, proliferation and differentiation capacities, cytokine secretory functions, surface markers, apoptosis, cell cycles, SA-β-gal staining, and gene expression levels in ADSCs from the two mice groups. Ultrastructurally, ADSCs from 20-month-old mice exhibited some senescence-associated changes, such as increased lipid droplets. The growth curve revealed that 20M ADSCs had less proliferative abilities than 1M ADSCs. Currently, the identification of senescent cells relies on a combination of multiple markers. Based on previous studies, Di Micco summarized senescence biomarkers, including the accumulation of p21 and p16 [44], in which p16 is the best-characterized senescence marker [45]. In this study, we found that 20M ADSCs expressed elevated p16, p19, and p21 levels compared to 1M ADSCs.

Cellular senescence is a state of irreversible cell cycle arrest [46]. Flow cytometry revealed that 20M ADSCs were significantly arrested at the G0 phase with more apoptotic cells than 1M ADSCs. We found that ADSCs from aged groups had some senescence-associated changes in ultrastructure, proliferation, gene expression, and cell cycle.

So could we conclude that the senescence of individuals is consistent with that of ADSCs? Maybe not. We took it a step further and explored more senescent parameters of ADSCs from aged donors. SA-β-gal was the first and most widely used biomarker of senescence while SA-β-gal-positive cells exhibited increased cell sizes which senescent cells exhibiting increased cell sizes [47, 48]. We found that primary 1M and 20M ADSCs did not express SA-β-gal and exhibited normal morphologies, while the expression levels of SA-β-gal were elevated and with flat morphologies after serial passaging. In the cytokine array, IL-6, PF-4, CXCL16, which ADSCs could secrete [28, 29], were significantly diminished in 20M ADSCs. Moreover, VEGF, an ADSC-secreted molecule [49], was found to be downregulated. Components of the senescence-associated secretory phenotype (SASP), including IL-6, were decreased in the culture supernatant of 20M ADSCs compared to 1M ADSCs, indicating that senescence phenotypes are highly heterogeneous and may differ depending on the cell type. The cytokine array revealed that elevated expression levels of GM-CSF, IL-4, IL-13, IL-17, CCL3, and CCL25 could be SASP profiles for ADSCs from aged donors. In addition, there were several differences between 1M and 20M ADSCs regarding the expression of surface markers and differentiation capacities. We found that 20M ADSCs had elevated CD105 expression levels and suppressed osteogenic as well as adipogenic differentiation capacities.

The molecular mechanisms associated with these phenotypes were also investigated. Various key age-related pathways have been reported, including insulin and IGF-1 signaling, whose multiple targets are the FOXO family of transcription factors and the PI3K/Akt/mTOR, which are also involved in aging [50–52]. Naishun Liao reported that antioxidant treatment can efficiently prevent the ADSC dysfunction and preserve cell functions after long-term passaging, providing a practical strategy to facilitate ADSC-based therapies [53]. A recent single-cell transcriptomic analysis of ADSCs from old donors revealed several enriched pathways, including CXCR4 signaling, implying that *p16*, *IL-6*, and *Cxcl1* are involved in senescence [54].

Our RNA-seq analysis revealed that some transcriptome changes associated with genes involved in age-related variations, such as *Lef1*, *Lingo2*, and *Dxd3y*. One of the major differences may stem from the upregulated genes because of the quantity gap. DEGs were significantly associated with inflammatory response, chemotaxis, chemokine activity, and chemokine, MAPK, PI3K-Akt signaling pathways. The Hippo signaling pathway and biological processes are also associated with aging, such as the anti-aging pathways AMP-activated protein kinase (AMPK) and sirtuin (SIRT) pathways [55], in which the downregulated DEGs were enriched. KEGG enrichment analysis and GSEA revealed that expression levels of genes associated with chemokine signaling pathway were altered. Moreover, bioinformatic gene network analysis of chemokine-related DEGs showed their association with the CCL7-CCL2-CCR2 axis. Integrated analyses of RNA-seq showed that *Lef1*, which plays a role in the Wnt signaling pathway, Hippo signaling pathway along with CCL7-CCL2-CCR2 axis may be involved in ADSCs age-related changes. Furthermore, qRT-PCR showed that expression levels of *Ccl7*, *Ccl2*, and *Ccr2* in ADSCs were elevated, confirming that CCL7-CCL2-CCR2 axis is the key mechanism underlying age-related changes.

## Conclusion

We investigated the characteristics of 20M ADSCs and verified the influence of senescence on the proliferation and differentiation of ADSCs in vitro. This study provides a theoretical basis for clinical applications of autologous ADSCs transplantation. ADSCs from old donors exhibited some changes, such as natural aging. Moreover, ADSCs can stand long-term cryopreservation with a high survival rate after resuscitation, without a significant impact on their proliferative and differentiation abilities [56, 57]. Therefore, we suggest that ADSCs should be cryopreserved in youth with a minimum number of passages to make autologous transplantation work better for senile diseases. Furthermore, our findings show that CCL7-CCL2-CCR2, a novel aging-associated regulatory axis, is a potential target for gene therapy to alleviate senescence in ADSCs.

## Supplementary Information

**Additional file 1.** Supporting Information Table S1. List of primers in qRT-PCR. Supporting Information Table S2. Targets of cytokine array. Supporting Information Table S3. RIN value of samples. Supporting Information Table S4. The quality control of RNA-seq data. Supporting Information Table S5. Alignment statistics resulted in RNA-seq data. Supporting Information Table S6. FPKM value of ADSCs in different growth stage. Supporting Information Table S7. Genes enriched in chemokine signaling pathway. Supporting Information Table S8. Top ranking neighbors in the expanded NTA. Supporting Information Fig. S1 Overall of cytokine expression levels. Supporting Information Fig. S2 Gating strategies of mADSCs surface marker expression profile analysis by flow cytometry. Supporting Information Fig. S3 RNA degradation was monitored using the agarose gels. Supporting Information Fig. S4 PANTHER pathway enrichment analysis of overall DEGs. Supporting Information Fig. S5 The top 5 of GO BP categories.

## Data Availability

All relevant data are within the paper and its online supplementary information files.

## Abbreviations

ADSCs: Adipose-derived stem cells
RNA-seq: Transcriptome sequencing
SA-β-gal: Senescence-associated β-galactosidase
SASP: Senescence-associated secretory phenotype
DEGs: Differentially expressed genes
NES: Normalized enrichment score
