# Supplementary material for: Age-related alteration in characteristics, function, and transcription features of ADSCs
Source: Stem Cell Res Ther. 2021 Aug 23;12:473. doi: 10.1186/s13287-021-02509-0 (PMC8383427; doi:10.1186/s13287-021-02509-0)
Supplement: Supplementary file 1 — Additional file 1. Supporting Information Table S1. List of primers in qRT-PCR. Supporting Information Table S2. Targets of cytokine array. Supporting Information Table S3. RIN value of samples. Supporting Information Table S4. The quality control of RNA-seq data. Supporting Information Table S5. Alignment statistics resulted in RNA-seq data. Supporting Information Table S6. FPKM value of ADSCs in different growth stage. Supporting Information Table S7. Genes enriched in chemokine signaling pathway. Supporting Information Table S8. Top ranking neighbors in the expanded NTA. Supporting Information Fig. S1 Overall of cytokine expression levels. Supporting Information Fig. S2 Gating strategies of mADSCs surface marker expression profile analysis by flow cytometry. Supporting Information Fig. S3 RNA degradation was monitored using the agarose gels. Supporting Information Fig. S4 PANTHER pathway enrichment analysis of overall DEGs. Supporting Information Fig. S5 The top 5 of GO BP categories. [file 13287_2021_2509_MOESM1_ESM.pdf]

Supporting Information Table S1. List of primers in qRT-PCR.

Gene	Forward	Reverse
p16	CGAACTCTTTCGGTCGTACCC	CGAATCTGCACCGTAGTTGAGC
p19	ATGGGTCGCAGGTTCTTGGT	GTAGTGGGGTCCTCGCAGTT
p21	TCCAGACATTCAGAGCCACA	CGAAGAGACAACGGCACACT
Ddx3y	GGAAGTAGCCGTGGACGTT	TCCATAGCCACCTCCACCA
S100a9	TCTGTGACTCTTTAGCCTTGAGCA	AGGGTGTCCTTCCTTCCTAGA
S100a8	GTCCTCAGTTTGTGCAGAATATAAA	GCCAGAAGCTCTGCTACTCC
Ngp	GCCACTCCGCCTTCTAGTC	TGCAATTTCTCTCCTCCCCAT
Chil3	ACCTGCCCCGTTCAGTG	GTCTTTCTCCACAGATTCTTCCTCA
Lef1	CTTCTCTGTCCCGATGGCAG	CATGTACGGGTCGCTGTTCA
Mcpt1	AGCTGGAGCTGAGGAGATTA	GTCCTCAGAACCTCTGTCCG
Dthd1	ACCCTCCTATTTCAACAGGTCC	GCCACTCTGGACAGTTCTCA
Ajap1	GCAAGCAAGGTCTGAGGCTG	CATGGAGCTGAGTCCTAAAAGC
Kcnmb4	AAGTGCTCCTATATCCCGCC	CCTCTGGTCTCTGATGCTGAT
Myc	GTTGGAAACCCCGCAGACA	CGTCGCAGATGAAATAGGGC
Axin2	GCGCTTTGATAAGGTCCTGG	TCATGTGAGCCTCCTCTCTTT
Taz	GAAGTTGATGCGTTGGACCC	TCCTACCCATTTGAAGCGCA
Yap1	GGGAGCAAGCCATGACTCAG	CCTCTGGTTCATGGCAAAACG
Ccl7	GTCTGCCAGCTCTCACTGAA	GCATTGGGCCCATCTGGTTG
Ccl2	CCCAAAGAAGCTGTAGTTTTTGTC	GACCTTAGGGCAGATGCAGTT
Ccr2	GCAAAGACCAGAAGAGGGCATT	GTATGCCGTG GATGAACTGA GG
β-actin	TGCTGTCCCTGTATGCCTCT	TTGATGTCACGCACGATTTC

Supporting Information Table S2. Targets of cytokine array.

A	B	C	D	E	F	G	H	I	J	K	L	M	N
Pos	Pos	Neg	Neg	BLANK	Axl	BLC	CD30 L	CD30 T	CD40	CRG-2	CTACK	CXCL16	Eotaxin
Pos	Pos	Neg	Neg	BLANK	Axl	BLC	CD30 L	CD30 T	CD40	CRG-2	CTACK	CXCL16	Eotaxin
Eotaxin-2	Fas Ligand	Fractalkine	GCSF	GM-CSF	IFN γ	IGFBP-3	IGFBP-5	IGFBP-6	IL-1a	IL-1 beta	IL-2	IL-3	IL-3 Rb
Eotaxin-2	Fas Ligand	Fractalkine	GCSF	GM-CSF	IFN γ	IGFBP-3	IGFBP-5	IGFBP-6	IL-1a	IL-1 beta	IL-2	IL-3	IL-3 Rb
IL-4	IL-5	IL-6	IL-9	IL-10	IL-12 p40/p70	IL-12 p70	IL-13	IL-17	KC	Leptin R	Leptin	LIX	L-Selectin
IL-4	IL-5	IL-6	IL-9	IL-10	IL-12 p40/p70	IL-12 p70	IL-13	IL-17	KC	Leptin R	Leptin	LIX	L-Selectin
Lymphotacti	MCP1	MCP-5	M-CSF	MIG	MIP-1α	MIP-1γ	MIP-2	MIP-3β	MIP-3α	PF-4	P-Selectin	RANTES	SCF
Lymphotacti	MCP1	MCP-5	M-CSF	MIG	MIP-1α	MIP-1γ	MIP-2	MIP-3β	MIP-3α	PF-4	P-Selectin	RANTES	SCF
SDF-1α	TARC	TCA-3	TECK	TIMP-1	TNFα	sTNF RI	sTNF RII	TPO	VCAM-1	VEGF	BLANK	BLANK	Pos
SDF-1α	TARC	TCA-3	TECK	TIMP-1	TNFα	sTNF RI	sTNF RII	TPO	VCAM-1	VEGF	BLANK	BLANK	Pos

Supporting Information Table S3. RIN value of samples.

Sample	Concentration (ng/μL)	Volume(μL)	Total(μg)	OD260/280	OD260/230	28S/18S	RIN value
1M-1	154	32	4.928	1.794	1.468	1.5	9.1
1M-2	315	32	10.08	1.925	1.805	1.7	9.3
1M-3	184	32	5.888	1.819	1.658	1.9	9.9
20M-1	416	32	13.312	1.944	0.832	2	9.6
20M-2	188	32	6.016	1.924	1.716	1.8	9.9
20M-3	280	32	8.96	1.925	1.627	1.9	10

Supporting Information Table S4. The quality control of RNA-seq data

Sample	Raw reads	Clean reads	Base pairs/G	Q20/%	Q30/%	GC/%
1M ADSCs	39151087	38131656	11.44	97.38	93.2	51.67
20M ADSCs	33514957	32718403	9.82	97.53	93.5	51.79

Supporting Information Table S5. Alignment statistics resulted in RNA-seq data

Sample	Total reads	Total mapped reads	Total mapped/%	Proper maped reads	Proper maped/%
1M ADSCs	76263312	72836628	95.51	67382202	88.35
20M ADSCs	65436806	62419338	95.39	57754308	88.26

Supporting Information Table S6. FPKM value of ADSCs in different growth stages

RPKM value	0-1	1-5	5-10	10-100	>100	Total
1M ADSCs	10435(47.31%)	4606(20.88%)	2438(11.05%)	4132(18.73%)	445(2.01%)	22056
20M ADSCs	10315(46.43%)	4841(21.79%)	2481(11.17%)	4111(18.50%)	468(2.10%)	22216

Supporting Information Table S7. Genes enriched in chemokine signaling pathway.

Symbol	Gene Name	Entrez Gene	Log_2FC[a]
Adcy2	adenylate cyclase 2	210044	2.1213
Adcy5	adenylate cyclase 5	224129	2.5353
Adcy8	adenylate cyclase 8	11514	2.3352
Ccl11	chemokine (C-C motif) ligand 11	20292	4.7095
Ccl12	chemokine (C-C motif) ligand 12	20293	10.0252
Ccl2	chemokine (C-C motif) ligand 2	20296	2.3461
Ccl3	chemokine (C-C motif) ligand 3	20302	9.5589
Ccl4	chemokine (C-C motif) ligand 4	20303	8.6995
Ccl6	chemokine (C-C motif) ligand 6	20305	8.5618
Ccl7	chemokine (C-C motif) ligand 7	20306	2.325
Ccl8	chemokine (C-C motif) ligand 8	20307	5.8505
Ccl9	chemokine (C-C motif) ligand 9	20308	3.3536
Ccr1	chemokine (C-C motif) receptor 1	12768	5.5111
Ccr2	chemokine (C-C motif) receptor 2	12772	7.8947
Ccr5	chemokine (C-C motif) receptor 5	12774	8.785
Cx3cr1	chemokine (C-X3-C motif) receptor 1	13051	2.0216
Cxcl1	chemokine (C-X-C motif) ligand 1	14825	2.7865
Cxcl11	chemokine (C-X-C motif) ligand 11	56066	5.4369
Cxcl12	chemokine (C-X-C motif) ligand 12	20315	2.2782
Cxcl13	chemokine (C-X-C motif) ligand 13	55985	6.27
Cxcl14	chemokine (C-X-C motif) ligand 14	57266	6.3108
Cxcl2	chemokine (C-X-C motif) ligand 2	20310	5.9128
Cxcl3	chemokine (C-X-C motif) ligand 3	330122	5.9128
Cxcl5	chemokine (C-X-C motif) ligand 5	20311	8.278

Cxcr2	chemokine (C-X-C motif) receptor 2	12765	6.1025
Cxcr4	chemokine (C-X-C motif) receptor 4	12767	5.0853
Dock2	dedicator of cyto-kinesis 2	94176	6.9467
Elmo1	engulfment and cell motility 1	140580	2.5818
Fgr	FGR proto-oncogene, Src family tyrosine kinase	14191	8.0866
Gng4	guanine nucleotide binding protein (G protein), gamma 4	14706	2.7035
Gngt2	guanine nucleotide binding protein (G protein), gamma transducing activity polypeptide 2	14710	2.1088
Grk3	G protein-coupled receptor kinase 3	320129	8.4777
Ncf1	neutrophil cytosolic factor 1	17969	4.9395
Pf4	platelet factor 4	56744	9.6067
Pik3cg	phosphatidylinositol-4,5-bisphosphate 3-kinase catalytic subunit gamma	30955	4.3968
Pik3r6	undefined	104709	5.6944
Plcb2	phospholipase C, beta 2	18796	9.4233
Ppbp	pro-platelet basic protein	57349	6.6804
Prex1	phosphatidylinositol-3,4,5-trisphosphate-dependent Rac exchange factor 1	277360	2.0495
Prkcb	protein kinase C, beta	18751	5.1936
Rac2	Rac family small GTPase 2	19354	10.5002
Vav1	vav 1 oncogene	22324	9.5589
Was	Wiskott-Aldrich syndrome	22376	4.8434

Average $\log_2$ fold change of most highly expressed genes set >2; Positive number is upregulated and negative is downregulated versus 1M ADSCs. Abbreviations: FC, fold change.

Supporting Information Table S8. Top ranking neighbors in the expanded NTA.

Gene Symbol	Random Walk Probability
Fancd2	6.73E-02
ACKR2	3.06E-02
Cbl	1.67E-02
Hnrnpa0	1.55E-02
CCL2	1.12E-02

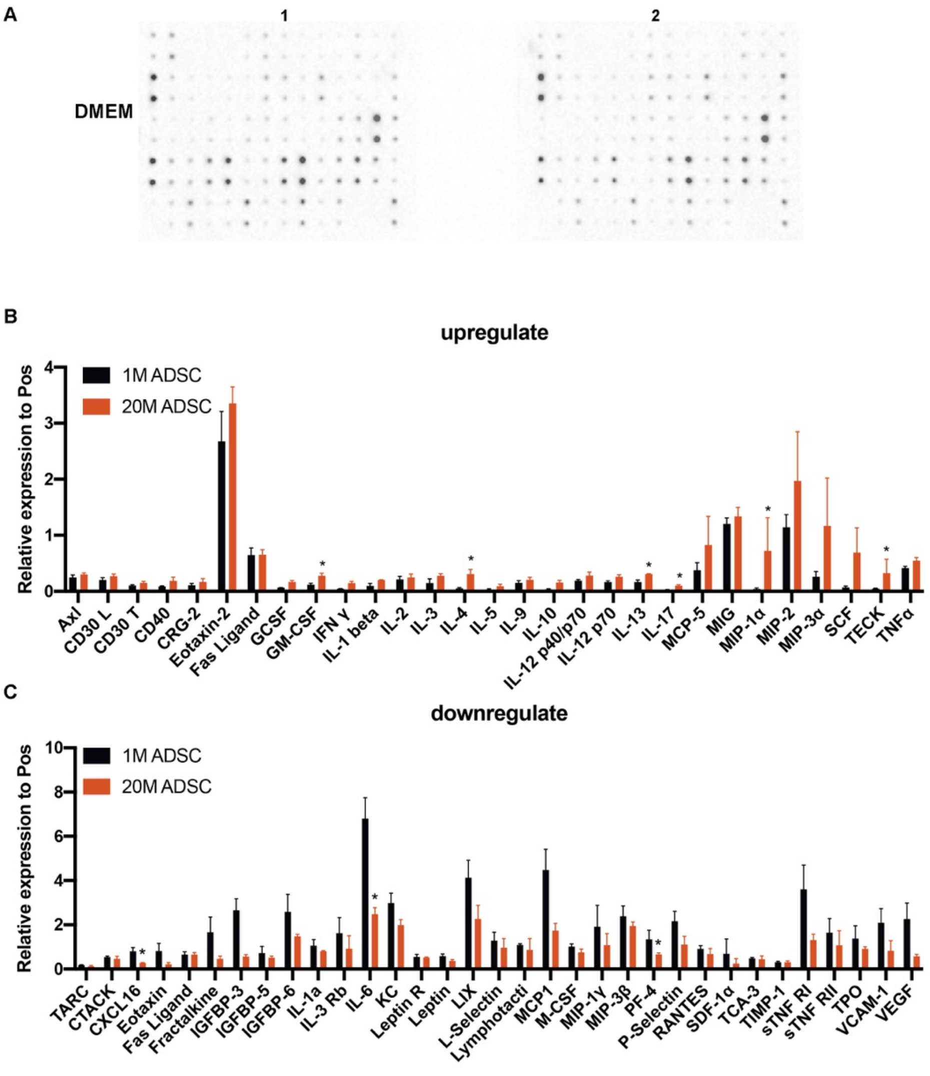

Supporting Information Fig.S1 Overall of cytokine expression levels.

A The DMEM membrane**. B** Up-regulated cytokines in 1M ADSCs were the controls**.** ∗, $p < 0.05$. **C** Down-regulated cytokines in 1M ADSCs were the controls**.** ∗, $p < 0.05$.

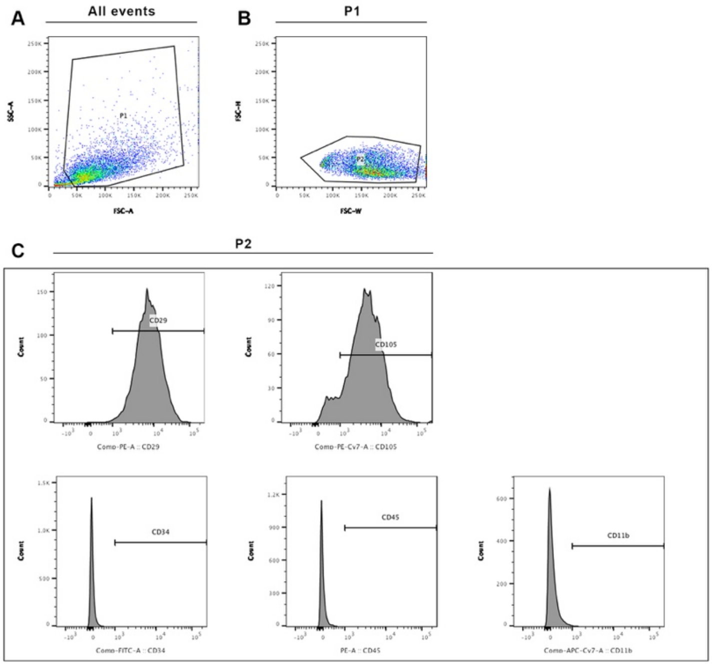

Supporting Information Fig.S2 Gating strategies of mADSCs surface marker expression profile analysis by flow cytometry

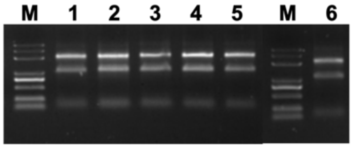

Supporting Information Fig.S3 RNA degradation was monitored using the agarose gels

M, marker. 1-3, 1M ADSCs. 4-6, 20M ADSCs.

A

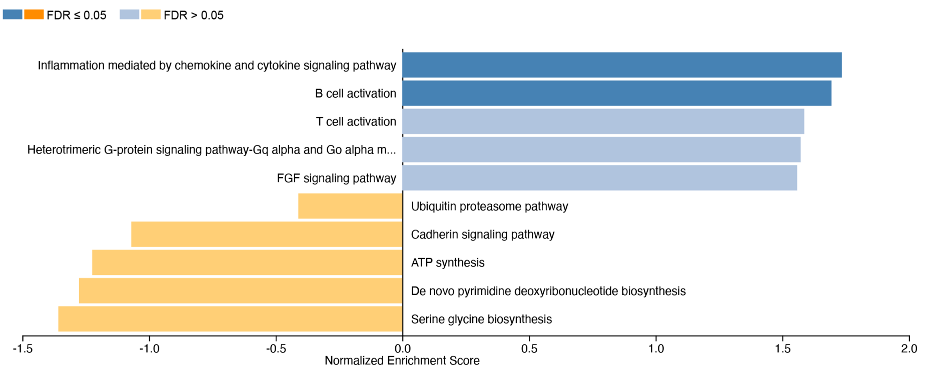

B

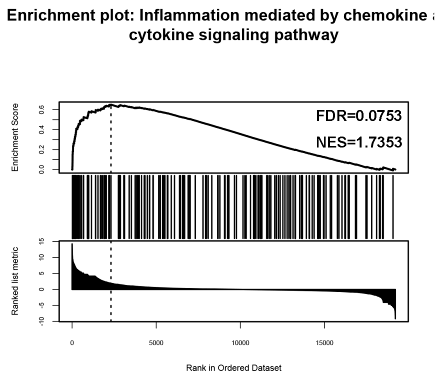

Supporting Information Fig.S4 PANTHER pathway enrichment analysis of overall DEGs.

A Bar chart showing the top 5 positive and negative related categories. **B** Enrichment plot of GSEA in significance level at FDR < 0.05. NES, normalized enrichment score. FDR, false discovery rate.

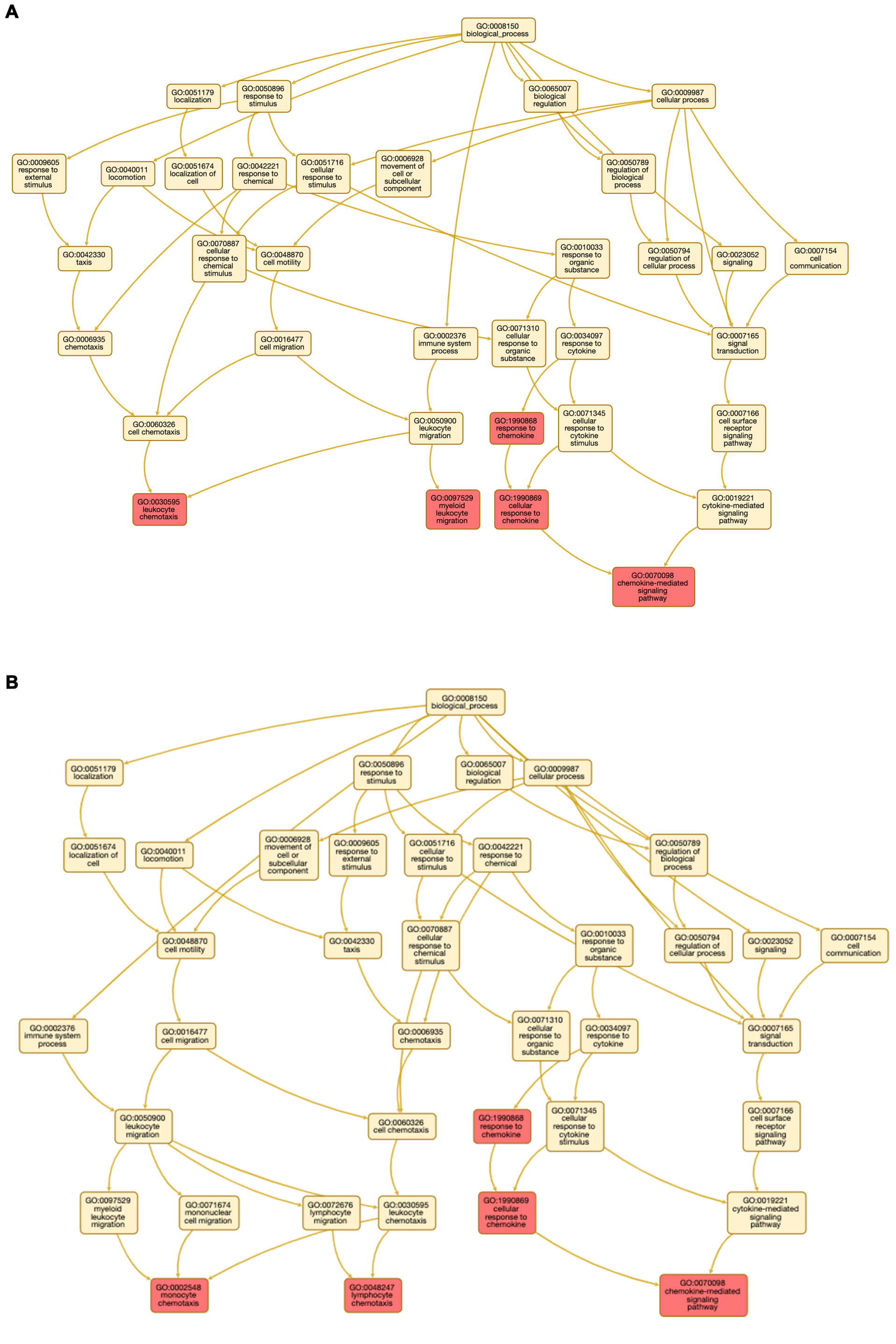

Supporting Information Fig.S5 The top 5 of GO BP categories

A Top 5 of GO BP categories in which all seeds and top ranking neighbors in the expanded sub-network were enriched**. B** Top 5 of GO BP categories in which *Ccl7, Ccl2,* and *Ccr2* were enriched**.**
